# Clinical-pharmacological drug information center of Hannover Medical School: experiences and analysis from a tertiary care university hospital

**DOI:** 10.1038/s41598-022-24005-y

**Published:** 2022-11-12

**Authors:** Johannes Heck, Dirk O. Stichtenoth, Ruxandra Sabau, Christoph Schröder, Stefan Engeli, Thorben Pape, Nina O’Connell, Carsten Schumacher, Olaf Krause, Felix Koop

**Affiliations:** 1 Institute for Clinical Pharmacology, Hannover Medical School, Carl-Neuberg-Str. 1, 30625 Hannover, Germany; 2 Drug Commissioner of Hannover Medical School (Dirk O. Stichtenoth); Head of Pharmacovigilance of Hannover Medical School (Christoph Schröder), Hannover, Germany; 3 Institute for Pharmacology, University Medicine Greifswald, Greifswald, Germany; 4 Center for Clinical Trials, Hannover Medical School, Hannover, Germany; 5 Institute for General Practice and Palliative Care, Hannover Medical School, Hannover, Germany; 6 Center for Medicine of the Elderly, DIAKOVERE Henriettenstift, Hannover, Germany

**Keywords:** Adverse effects, Drug therapy

## Abstract

Drug information centers (DICs) are institutions dedicated to provide objective, independent, and up-to-date information on drugs and their rational use. To overcome the lack of recent DIC reports from central Europe, we analyzed all queries (n = 594) submitted to the DIC run by the Institute for Clinical Pharmacology of Hannover Medical School between October 2018 and April 2022. Approximately one in three queries (31.1%; 185/594) was submitted by internists. 82.8% (492/594) of the queries were patient-specific, while the remaining 17.2% (102/594) were general queries. Adverse drug reactions (ADRs), indications/contraindications, and pharmacodynamic interactions (PDIs) represented the three most frequently addressed query categories, being involved in 44.8% (266/594), 43.3% (257/594), and 34.3% (204/594) of all queries, respectively (assignment of more than one category per query was possible). As compared to general queries, patient-specific queries were statistically significantly more often related to ADRs, PDIs, and pharmacokinetic interactions (PKIs) (ADRs: 35.3% vs. 46.7%, *P* = 0.034; PDIs: 14.7% vs. 38.4%, *P* < 0.001; PKIs: 20.6% vs. 31.5%, *P* = 0.028). To demonstrate the complexity of queries submitted to the clinical-pharmacological DIC, we present and comment on an illustrative selection of queries.

## Introduction

Drug information centers (DICs) are defined as “institution[s] dedicated to provide objective, independent and current information on drugs and their use, and communicate to the different categories of users for better understanding and benefit of patients”^1^. DICs play a key role in the dissemination of drug information (DI) to healthcare professionals all over the globe, thus promoting the rational use of drugs^1,2^. The first DIC was inaugurated at the University of Kentucky in the United States in 1962^3,4^, and numerous DICs followed in the United States and Europe throughout the 1960s to the 1980s^1,4–6^. Today, DICs are present in most countries worldwide^1^, also in emerging countries such as India^7–11^, Nepal^12^, Brazil^13^, Uganda^14^, or Ethiopia^15–17^.

Staff composition and modus operandi of DICs differ markedly between countries. In the United States, for example, DICs are typically led by pharmacists and responses to DI queries are generally provided by telephone^18,19^. European DICs are frequently headed by a clinical pharmacologist or similarly qualified physician, and are run jointly by physicians (usually clinical pharmacologists) and pharmacists^5,6,19,20^. However, there also exist numerous pharmacist-led DICs in Europe. Owing to their affiliation with university hospitals, European DICs are often assisted by visiting physicians and medical students^21^. European DICs generally provide written responses in addition to verbal communication with inquirers^21^.

While several reports from emerging countries^7–11,13–17^ and Scandinavia^5,19,22–25^ have been published in recent years, there is a considerable lack of studies on DICs from central Europe, in particular from Germany. To the best of our knowledge, the most recent data from Germany on clinical pharmacologist-led DICs which provide DI to other healthcare professionals stem from Tröger and Meyer from 2000^26^ and from Schwarz and colleagues from 1999^27^. To close this information gap of more than 20 years, we analyzed all queries submitted to the DIC operated by the Institute for Clinical Pharmacology of Hannover Medical School between October 2018 and April 2022. We compared the frequency and characteristics of DI queries with previous studies from Germany and other countries and investigated potential differences between patient-specific and general queries. In addition, we present and comment on an illustrative and educational selection of clinically complex queries answered by our clinical-pharmacological DIC during the study period.

## Methods

### Ethics approval

This study adheres to the Declaration of Helsinki (1964) and its later amendments (latest version 2013). Due to anonymous data analysis, the Ethics Committee of Hannover Medical School waived a formal ethics vote.

### Modus operandi of the clinical-pharmacological drug information center of Hannover Medical School

The clinical-pharmacological DIC of Hannover Medical School was established at the Institute for Clinical Pharmacology in 1994. As a specialist pharmacotherapeutic consultation service, the clinical-pharmacological DIC is available to physicians and other healthcare professionals (e.g., dentists, nurses) working at Hannover Medical School or affiliated academic teaching hospitals free of charge. Occasionally, the clinical-pharmacological DIC also answers queries from physicians working in private practice, for example, if they are the ambulatory physicians of patients who were treated at Hannover Medical School. Of note, the hospital pharmacy of Hannover Medical School provides its own drug information service, which operates independently from the clinical-pharmacological DIC and which is specialized on pharmaceutical questions. A Computerized Provider Order Entry and Clinical Decision Support System (CPOE-CDSS) is not routinely available at Hannover Medical School.

The modus operandi of the clinical-pharmacological DIC is laid down in standard operating procedures (SOPs). The clinical-pharmacological DIC is operated by two senior physicians (one clinical pharmacologist (DOS) and one internist (CS)), one resident in clinical pharmacology (JH), one clinical pharmacist with specialty training in drug information (according to the curriculum of the German Federal Chamber of Pharmacists (*Fachapothekerin für Arzneimittelinformation gemäß Curriculum der Bundesapothekerkammer*); RS), and one secretary. Queries are submitted to the clinical-pharmacological DIC by healthcare professionals in written form via the hospital information system SAP (Systems, Applications, and Products in Data Processing, Walldorf, Germany) or via e-mail. Queries are attended to by JH or RS, with occasional support from visiting physicians or medical students. If relevant clinical information is missing (e.g., information about the patient’s medication or comorbidities), the inquiring healthcare professional is contacted via telephone or e-mail. Subsequently, a comprehensive literature review is conducted, taking advantage of the following references (non-exhaustive list): summaries of product characteristics (SmPCs), medical databases (e.g., PubMed, Cochrane Library), current editions of standard textbooks of (clinical) pharmacology (e.g., Stockley’s Drug Interactions, Goodman & Gilman’s The Pharmacological Basis of Therapeutics, The Renal Drug Handbook), and drug information and interaction programs (e.g., UpToDate (Wolters Kluwer N.V., Alphen aan den Rijn, The Netherlands), AiDKlinik (Dosing GmbH, Heidelberg, Germany), mediQ (Psychiatrische Dienste Aargau AG, mediQ Kompetenzzentrum für Medikamentensicherheit, Windisch, Switzerland)). A written response is drafted, and it is discussed with and countersigned by a senior physician (DOS or CS). The finalized response is sent to the inquiring healthcare professional via SAP or e-mail within one to three workdays. In urgent cases, a preliminary answer is communicated by telephone.

All queries and corresponding answers are saved in a password-protected Microsoft Access 2016 database (Redmond, Washington, USA) which is only accessible to registered users, i.e., staff of the clinical-pharmacological DIC. In addition, paper-based documents, such as printed scientific articles which were used to answer queries, handwritten notes, protocols of telephone communications, answer drafts, and so forth, are stored in folders which are kept in locked rooms in locked cupboards (separate sets of keys for rooms and cupboards). With the exception of age and sex, any patient-related data is deleted prior to saving the queries and corresponding answers in the electronic database and in the paper-based documentation.

All queries and corresponding answers are presented and discussed at weekly pharmacotherapeutic colloquia. These colloquia serve as additional internal quality assurance and are accredited by the Medical Association of Lower Saxony (*Ärztekammer Niedersachsen*) as continuing medical education (CME) seminars for physicians. Besides, medical students in the practical year (*Praktisches Jahr*—i.e., the final year of the medical curriculum in Germany) are encouraged to participate in pharmacotherapeutic colloquia as part of their medical training at Hannover Medical School.

Of note, the clinical-pharmacological DIC is not the only service provided by the Institute for Clinical Pharmacology. Other tasks comprise pharmacovigilance in clinical trials, teaching of medical students, interdisciplinary ward rounds, clinical research, and the office of the Drug Commissioner of Hannover Medical School.

### Data acquisition

For the purpose of this study, all queries submitted to the clinical-pharmacological DIC between October 2018 and April 2022 were analyzed. For each query, the following parameters were assessed:

- origin of the query: Hannover Medical School; academic teaching hospital; private practice; other
- type of query: patient-specific (i.e., queries referring to individual patients) or general (i.e., queries of broader pharmacological interest or queries referring to larger patient populations)
- age and sex of the patient (only for patient-specific queries)
- the medical specialty of the inquiring healthcare professional
- query categories (assignment of more than one category per query was possible): adverse drug reaction (ADR); indication/contraindication; posology/dose adjustment (e.g., due to renal or hepatic insufficiency); therapeutic drug monitoring (TDM); pharmacogenetics; pharmacodynamic interaction (PDI); pharmacokinetic interaction (PKI); pregnancy and breastfeeding; pharmacotherapy in the elderly (i.e., patients ≥ 65 years of age); other.

Of note, the category “pharmacotherapy in the elderly” was not automatically assigned to all queries concerning patients of chronological age of ≥ 65 years, but only to queries explicitly inquiring about drug specifics in advanced age.

### Statistical analysis

Descriptive statistical techniques were used to summarize the data. Quantitative variables are depicted as means ± standard deviations (SDs) and additionally as medians with interquartile ranges (IQRs) for data that were not normally distributed. For categorical variables, absolute and relative frequencies are presented. Differences between general and patient-specific queries regarding involved query categories were analyzed with Pearson’s Chi-squared test or Fisher’s exact test (the latter was applied if any of the four cells of a 2 × 2 table had less than five observations). *P *values < 0.05 were considered statistically significant. All statistical analyses were conducted with Microsoft Excel 2019 (Redmond, Washington, USA) and IBM SPSS Statistics for Windows, version 28 (Armonk, New York, USA).

## Results

### Number, type, and origin of queries submitted to the clinical-pharmacological drug information center

In total, 594 queries were submitted to the clinical-pharmacological DIC during the study period (i.e., 43 months). Of these, 82.8% (492/594) were patient-specific queries, while the remaining 17.2% (102/594) were general queries (Table 1). The vast majority of queries were submitted by physicians (96.1%; 571/594), and more than three-quarters of queries (75.6%; 449/594) came from healthcare professionals working at Hannover Medical School. On an aggregated level (i.e., without differentiation between subdisciplines of internal medicine, surgery, etc.), approximately one in three queries (31.1%; 185/594) came from internists. If subdisciplines of internal medicine, surgery, etc. were evaluated separately, psychiatrists, urologists, and trauma surgeons most frequently consulted the clinical-pharmacological DIC, submitting 10.6% (63/594), 9.3% (55/594), and 8.4% (50/594) of all queries, respectively (Supplementary Table 1).

**Table 1:** Characteristics of queries submitted to the clinical-pharmacological drug information center of Hannover Medical School between October 2018 and April 2022 (n = 594).

Variables	No. of submitted queries	%
**Type of query**		
Patient-specific	492	82.8
General	102	17.2
**Origin of query**		
Hannover Medical School	449	75.6
Academic teaching hospital	77	13.0
Private practice	18	3.0
Other^a^	50	8.4
**Profession of inquiring healthcare professionals**		
Physician	571	96.1
Dentist	3	0.5
Pharmacist	2	0.3
Other (e.g., medical student)	18	3.0
**Specialization of inquiring healthcare professionals**^b^		
Internal medicine	185	31.1
Psychiatry and psychosomatic medicine	85	14.3
Surgery	83	14.0
Urology	55	9.3
Pediatrics	27	4.5
Gynecology and obstetrics	24	4.0
Radiology and radiotherapy	20	3.4
General practice	19	3.2
Dermatology	18	3.0
Neurology	16	2.7
Other	44	7.4
Not documented	18	3.0

^a^Comprises queries from medical students not affiliated with Hannover Medical School, physicians working at laboratories or agencies (e.g., public health department), or retired physicians, among others.

^b^Data presented in aggregated form (i.e., without differentiation between subdisciplines of internal medicine, surgery, etc.); the full description of medical specialties is shown in Supplementary Table 1.

### Patient characteristics

**Figure 1:**
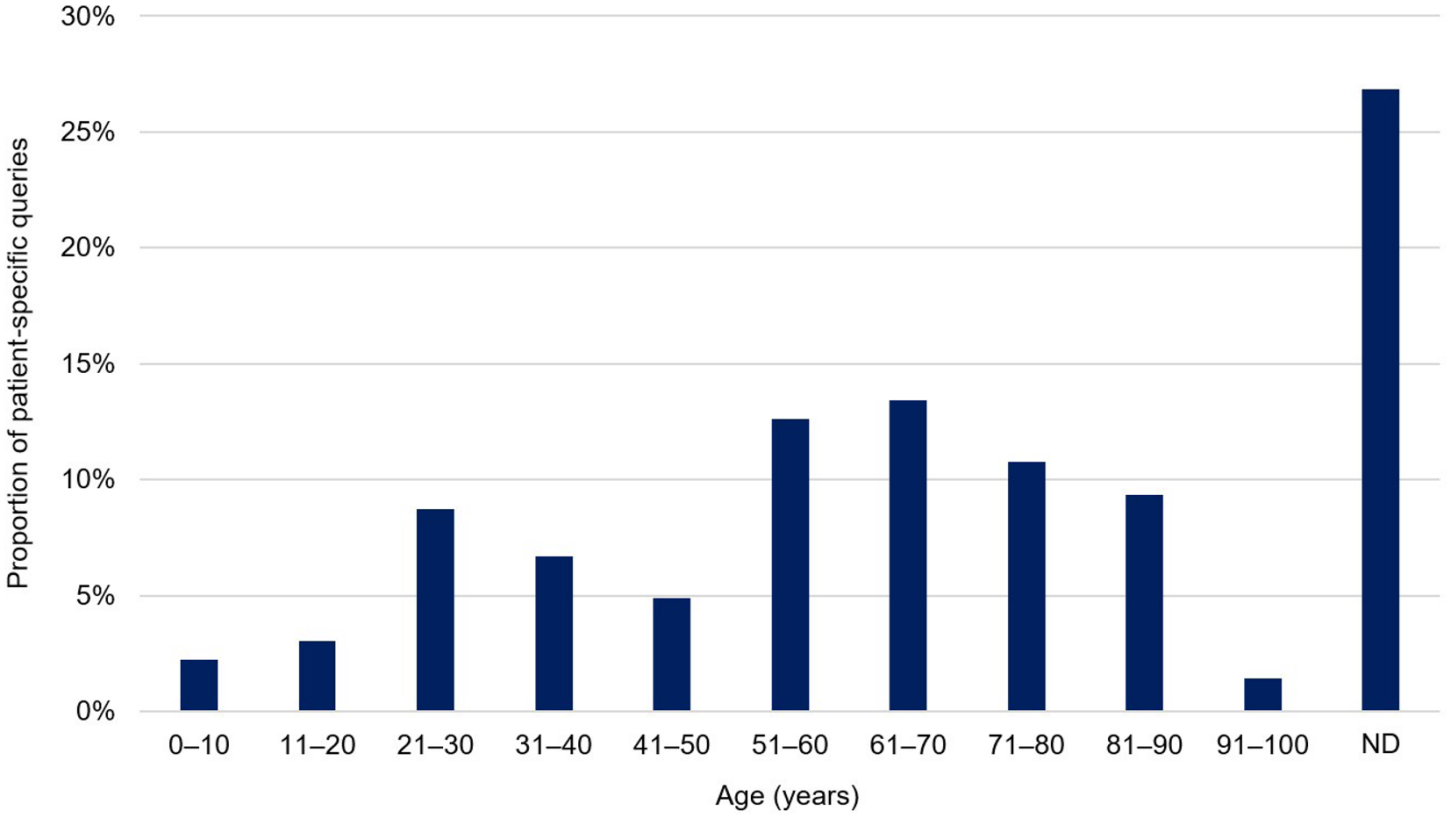
Age distribution in patient-specific queries (n = 492) submitted to the clinical-pharmacological drug information center of Hannover Medical School between October 2018 and April 2022. ND denotes not documented.

Age and sex of patients were documented in 73.2% (360/492) and 92.3% (454/492) of patient-specific queries, respectively. The mean age of patients in patient-specific queries was 55.6 ± 22.9 years (median 60 years, IQR 37 to 72 years, range 3 days to 100 years). 51.4% (253/492) of the patients were female (male: 40.9% (201/492); sex not documented: 7.7% (38/492)). As displayed in Fig. 1, patient-specific queries were most frequently related to patients aged between 61 and 70 years (13.4% (66/492) of patient-specific queries).

### Query categories

ADRs, indications/contraindications, and PDIs represented the three most frequent query categories, being involved in 44.8% (266/594), 43.3% (257/594), and 34.3% (204/594) of all queries, respectively (Table 2).

**Table 2:** Absolute and relative frequencies of query categories involved in queries submitted to the clinical-pharmacological drug information center of Hannover Medical School between October 2018 and April 2022. Assignment of more than one category per query was possible.

Query category	Total (n = 594)	Patient-specific queries (n = 492; 82.8%)	General queries (n = 102; 17.2%)	*P* value
Adverse drug reaction—no. (%)	266 (44.8)	230 (46.7)	36 (35.3)	**0.034**^**a**^
Indication/contraindication—no. (%)	257 (43.3)	215 (43.7)	42 (41.2)	0.640^a^
Posology/dose adjustment (e.g., due to renal or hepatic insufficiency)—no. (%)	138 (23.2)	117 (23.8)	21 (20.6)	0.487^a^
Therapeutic drug monitoring—no. (%)	63 (10.6)	55 (11.2)	8 (7.8)	0.319^a^
Pharmacogenetics—no. (%)	24 (4.0)	22 (4.5)	2 (2.0)	0.404^b^
Pharmacodynamic interaction—no. (%)	204 (34.3)	189 (38.4)	15 (14.7)	** < 0.001**^**a**^
Pharmacokinetic interaction—no. (%)	176 (29.6)	155 (31.5)	21 (20.6)	**0.028**^**a**^
Pregnancy and breastfeeding—no. (%)	21 (3.5)	16 (3.3)	5 (4.9)	0.383^b^
Pharmacotherapy in the elderly—no. (%)	36 (6.1)	33 (6.7)	3 (2.9)	0.147^a^
Other—no. (%)	84 (14.1)	56 (11.4)	28 (27.5)	** < 0.001**^**a**^

*P* values < 0.05 were considered statistically significant and are highlighted in bold.

^a^Pearson’s chi-squared test.

^b^Fisher’s exact test.

Absolute and relative frequencies of query categories, stratified by specializations of inquiring healthcare professionals, are shown in Supplementary Table 2. ADRs were most frequently inquired by physicians working in radiology/radiotherapy (55.0% (11/20) of all queries submitted by physicians from these two disciplines), while indications/contraindications and pharmacogenetics were most frequently referred to by psychiatrists (60.0% (51/85) and 10.6% (9/85) of all queries submitted by psychiatrists, respectively). Queries concerning posology and/or dose adjustments were most commonly submitted by dermatologists (44.4% (8/18) of all queries from dermatologists), whereas TDM was most frequently inquired by pediatricians (22.2% (6/27) of all queries from pediatricians). PDIs were involved in nearly two-thirds (65.5% (36/55)) of all queries from urologists, while PKIs were implicated in almost half (45.8% (11/24)) of all queries from gynecologists. Pharmacotherapy during pregnancy and breastfeeding was most frequently addressed by dermatologists (11.1% (2/18) of all queries from dermatologists), while pharmacotherapy in older people was referred to by approximately every fifth query (20.5% (17/83)) submitted by surgeons.

### Comparison between general and patient-specific queries

As compared to general queries, patient-specific queries were statistically significantly more often related to ADRs, PDIs, and PKIs (ADRs: 35.3% vs. 46.7%, *P* = 0.034; PDIs: 14.7% vs. 38.4%, *P* < 0.001; PKIs: 20.6% vs. 31.5%, *P* = 0.028) (Table 2). By contrast, the query category “other” was more often addressed by general queries as compared to patient-specific queries (27.5% vs. 11.4%, *P* < 0.001).

### Examples of clinically complex queries answered by the clinical-pharmacological drug information center

To illustrate the clinical complexity of DI queries submitted to and answered by the clinical-pharmacological DIC, we present and comment on an educational selection of ten queries (Table 3). Half of these cases were related to rare diseases (i.e., cases no. 1, 2, 7, 8, and 10), while one case each referred to toxicology (case no. 3), drug–drug interactions (case no. 4), pharmacotherapy in the elderly (case no. 5), pharmacotherapy during pregnancy (case no. 6), and TDM (case no. 9).

**Table 3:** Examples of queries answered by the clinical-pharmacological drug information center of Hannover Medical School between October 2018 and April 2022.

Case no.	Question	Answer	Comment
1	“An 84-year-old female patient suffering from mycosis fungoides (cutaneous T-cell lymphoma) is going to be treated with bexarotene. Since elevated serum triglyceride levels represent a frequent side effect of bexarotene, we plan to administer fenofibrate alongside bexarotene. Due to comorbid cardiovascular disease (not otherwise specified), the patient is already taking atorvastatin. According to the drug interaction program AiDKlinik, the co-administration of fenofibrate and atorvastatin may increase the risk of myopathy and rhabdomyolysis. How should we proceed?”	“The reported drug–drug interaction between fenofibrate and atorvastatin can be characterized as pharmacodynamic, leading to an addition of possible side effects. A switch of therapy to—for example—pravastatin (which does not interfere with the cytochrome P450 enzyme system) therefore does not offer advantages compared to atorvastatin. Two different strategies can be recommended: (i) prescription of fenofibrate together with a low dose of atorvastatin (20 mg/day) and regular monitoring of creatine kinase and transaminase levels; or (ii) monotherapy with atorvastatin and addition of fenofibrate only if serum triglyceride levels surpass 400 mg/dl, according to the algorithm proposed by Talpur and colleagues.” ^46^	Treatment with bexarotene frequently entails elevated serum triglyceride levels, which may necessitate discontinuation of bexarotene in order to reduce the risk of pancreatitis. According to the algorithm set forth by Talpur and co-workers, a temporary halt of bexarotene should be considered if serum triglyceride levels surpass 800 mg/dl. Prevention/treatment of hyperlipidemia allows for a continuous bexarotene therapy, resulting in a better treatment outcome with respect to cutaneous T-cell lymphoma. However, a stringent lipid-lowering therapy with statins and/or fibrates requires regular monitoring of muscle and liver enzymes to lower the risk of myopathy and rhabdomyolysis.
2	“A 69-year-old female patient with recurrent symptomatic episodes of hypoglycemia (blood glucose levels of 3.33 ± 1.41 mmol/l) is displaying hyperinsulinemia (> 1000 mU/l) and low C-peptide levels (2.27 ± 0.63 nmol/l). Further examinations were inconspicuous of insulinoma or other paraneoplastic hypoglycemia. Prior to the onset of the recurrent hypoglycemic episodes, there was a comprehensive change in the patient’s medication. Do you see a possible link between the patient’s (change in) medication and the debut of hyperinsulinemia/hypoglycemia? The possibility of a factitious disorder in the sense of exogenous insulin administration has been excluded. The patient’s current medication comprises mirtazapine 30 mg 0–0–1, metamizole 500 mg 0–0–1, pantoprazole 20 mg 1–0–0, simvastatin 30 mg 0–0–1, acetylsalicylic acid 100 mg 1–0–0, losartan 100 mg 1–0–0, moxonidine 0.5 mg 1–0–1, bisoprolol 10 mg 0–0–1, doxazosin 4 mg 1–0–1, hydrochlorothiazide 12.5 mg 1–0–0, lercanidipine 20 mg 1–0–0, clopidogrel 75 mg 1–0–0, and cholecalciferol 1000 IU 1–0–0.”	“Clopidogrel has been described to very rarely elicit drug-induced insulin autoimmune syndrome. Given the temporal relationship between the change in the patient’s medication and the onset of the hypoglycemic episodes, we suspect clopidogrel as the causative agent, and we suggest determining the patient’s insulin autoantibody titer.” ^47^	In the presented case, the insulin autoantibody titer of > 100 IU/ml confirmed the suspected diagnosis of clopidogrel-induced insulin autoimmune syndrome. Consequently, clopidogrel was discontinued. In addition, repetitive immunoadsorption was required to terminate the hypoglycemic episodes permanently. The case was published in the German journal *Der* *Internist.* ^62^
3	“A female patient was admitted to our clinic with hypoglycemia, abdominal pain, and high urine ketone levels. Under the assumption of the initial manifestation of type-1 diabetes mellitus, the patient was transferred to the intensive care unit where diabetes mellitus could rapidly be ruled out. Upon admission, acute kidney injury with creatinine levels of up to 3.47 mg/dl was observed but creatinine levels normalized already on the second day of hospitalization. Liver transaminases were slightly elevated; bilirubin levels were initially normal and then slightly elevated; the INR was inconspicuous. When explicitly asked about the intake of over-the-counter preparations, the patient stated that she was treating herself with spirulina (a dietary supplement of blue-green algae) and L-carnitine. Are you aware of any interactions between spirulina and L-carnitine that might explain the patient’s temporary organ dysfunctions upon hospital admission?”	“To the best of our knowledge, there are no clinically relevant interactions between spirulina and L-carnitine. However, the patient’s symptoms might be indicative of an intoxication with cyanobacterial toxins (e.g., microcystins). Contamination of over-the-counter algae preparations with cyanobacterial toxins is increasingly being recognized as a serious problem. Cyanobacterial toxins can exert pronounced hepatotoxic and neurotoxic effects. As no specific antidote is available, we recommend the discontinuation of the spirulina product along with symptomatic therapy.” ^48,49^	The risks of uncritical and medically unsupervised intake of dietary supplements are often underestimated by patients. In case of unclear diagnosis, it is prudent for the treating physician to thoroughly evaluate the patient’s medication including self-medication.
4	“One of our patients opposed treatment with metamizole, fearing the drug might interact with her co-medication. The patient cited an online open-access drug interaction checker which described metamizole as a potent inducer of CYP3A4. I am unaware of any CYP-inducing potential of metamizole and—considering the widespread use of metamizole in Germany—I doubt that this is of clinical relevance. What is your stance on the CYP-inducing potential of metamizole? Do you have access to any additional data that I may have overlooked?”	“Based on in vitro experiments, Saussele and colleagues reported in 2007 that metamizole was an inducer of CYP2B6 and CYP3A4. In the absence of clinical data, the authors stated that further studies were warranted to fully elucidate the clinical relevance of their results. Notwithstanding, some open-access drug interaction programs have listed metamizole as a potent inducer of the abovementioned CYP enzymes based on the results of Saussele et al. The SmPC of metamizole as well as standard reference works do not comment on any CYP-inducing potential of metamizole. As to date (i.e., 2020) no data from clinical studies are available, we consider the current knowledge insufficient to assume clinically relevant pharmacokinetic drug–drug interactions elicited by metamizole via induction of CYP3A4.” ^50,51^	One year after this query had been submitted to the clinical-pharmacological DIC, Bachmann et al. investigated CYP enzyme activity after metamizole treatment in twelve healthy male adults and detected a moderate induction of CYP2B6, CYP3A4, CYP2C9, and CYP2C19. This demonstrates that medical knowledge in general and clinical-pharmacological knowledge in particular are subject to rapid change, highlighting the importance of DICs in the provision of up-to-date drug information and treatment recommendations.
5	“A 75-year-old cachectic (BMI 16.9 kg/m^2^) patient with moderate dementia and paranoid schizophrenia is currently being treated in a department of psychiatry due to aggressive behavior. Ever since the patient’s new attending neurologist discontinued olanzapine, the patient’s condition deteriorated significantly with increasing aggression and unrest. The patient’s current medication comprises acetylsalicylic acid 100 mg 1–0–0–0, folic acid 5 mg 1–0–0–0, donepezil 10 mg 0–0–1–0, haloperidol 3 mg 1–1–1–0, pipamperone 40 mg 0–1–0–1, olanzapine 5 mg 1–0–1–2, lorazepam 1 mg 1–1–1–1, and enoxaparin SC. Could the medication be responsible for the recent worsening of the patient’s condition?”	“The co-prescription of three antipsychotic drugs and a benzodiazepine should be viewed critically in the case of a 75-year-old cachectic patient. Haloperidol (at a daily dose of ≥ 2 mg) and olanzapine (at a daily dose of ≥ 10 mg) are considered as potentially inappropriate medications for elderly people according to the PRISCUS list. Furthermore, lorazepam may elicit paradoxical reactions in older patients, including increased psychomotor activity and aggressiveness. If haloperidol as a potent first-generation antipsychotic (FGA) is required, the less potent FGA pipamperone may be discontinued. Also, it should be noted that the combination of haloperidol and donepezil can lead to QT_c_ interval prolongation. Considering the patient’s age, initiation of a proton pump inhibitor may reduce the risk of gastrointestinal bleeding under combined therapy with enoxaparin and acetylsalicylic acid. However, taking the risk of falling with ensuing intracerebral hemorrhage into account, the indication for enoxaparin and acetylsalicylic acid should be critically re-evaluated.” ^52,53^	Pharmacotherapy in elderly patients, particularly psychopharmacological therapy, poses a significant challenge to physicians. The co-prescription of several psychotropic drugs is often overlooked as a possible trigger of neuropsychiatric adverse events. A regular and critical review of the medication can reduce the number of medication-related problems. Tools such as the PRISCUS list can help identify medications that should be avoided in elderly people.
6	“A 21-year-old pregnant woman (ninth week of pregnancy) with multidrug-resistant tuberculosis is receiving an anti-infective therapy consisting of levofloxacin, bedaquiline, terizidone, clofazimine, amoxicillin–clavulanate, para-aminosalicylic acid (all PO), and meropenem IV. Besides, she is being treated with cholecalciferol PO. We plan to change para-aminosalicylic acid from PO to IV administration soon. Initially, the patient was also treated with amikacin IV. The planned total duration of the anti-infective therapy is 12 months. How do you evaluate the safety of the cited drugs during pregnancy and the hazards for the fetus?”	“None of the mentioned medications, except for amoxicillin–clavulanate and cholecalciferol (strict indication provided), are suitable for treatment during pregnancy. Of the cited drugs, we consider amikacin (which was already stopped) and clofazimine as the least safe medications during pregnancy. Considering that clofazimine is only approved for the treatment of leprosy, we suggest stopping it. If the benefits to the mother are expected to outweigh the risks to the child, the use of levofloxacin, bedaquiline, terizidone, meropenem, and para-aminosalicylic acid seems reasonable under the given circumstances. We suggest regular ultrasound diagnostics to monitor fetal health and development. The outcome of the pregnancy should be reported to the pharmacovigilance division of the Federal Institute for Drugs and Medical Devices (*Bundesinstitut für Arzneimittel und Medizinprodukte*).” ^54^	Many medications are inappropriate or are insufficiently evaluated during pregnancy. While the expected benefit to the mother can outweigh the risks to the child, a critical evaluation of medications during pregnancy or breastfeeding is indispensable in order to reduce the negative impact on the developing child to a minimum.
7	“A 55-year-old male patient is suffering from recurrent pituitary insufficiency after surgical removal of an atypical glioma of the third ventricle approximately twenty years ago. Currently, the patient is experiencing hypothermia requiring the application of heated blankets to maintain his body temperature. The patient’s medication comprises levothyroxine 25 µg 1–0–0, pantoprazole 40 mg 1–0–0, amlodipine 5 mg 1–0–0, ramipril 10 mg 1–0–0, lacosamide 200 mg 1–0–1, urapidil sustained release 30 mg 1–0–1, cotrimoxazole 480 mg three times per week, hydrocortisone IV, and fondaparinux 2.5 mg SC once daily. Which medication (e.g., clonidine, clomipramine, or cyproheptadine) would you recommend to treat the patient’s hypothermia?”	“To the best of our knowledge, no guidelines or randomized clinical trials regarding the use of clonidine, clomipramine, or cyproheptadine for the treatment of hypothermia are available. Notwithstanding, all of the cited drugs have been described in case reports. Clomipramine is suited for long-term therapy and has been described in various case reports as a feasible option; therefore, we suggest treatment with clomipramine as the first choice. It must be noted, however, that the co-administration of clomipramine and cotrimoxazole can prolong the QT_c_ interval and hence requires ECG examinations before and during therapy. We recommend a dosage of 35 mg clomipramine per day. Alternatively, the patient’s hypothermia may be treated with clonidine (e.g., 0.15 mg three times daily). Considering the already established antihypertensive therapy with amlodipine, ramipril, and urapidil, we suggest a careful dose titration of clonidine because of possible synergistic antihypertensive effects. Due to potential ADRs of cyproheptadine (e.g., anticholinergic side effects) and little practical experience of most physicians with this rarely used drug, we consider cyproheptadine as the third choice in the presented case.” ^55^	Periodic hypothermia can be a symptom of pituitary insufficiency and may require pharmacological therapy. In the absence of high-level evidence, our recommendation in the presented case was mostly based on expert opinion.
8	“A patient with acute porphyria is displaying a fungal infection in the shoulder region. Currently, the patient is being treated with caspofungin IV. The therapy is planned for a duration of three months; therefore, we would like to switch to oral treatment. Which oral antimycotic can be administered in lieu of caspofungin IV?”	“Antimycotics of choice for patients with acute porphyria are echinocandins (e.g., caspofungin), amphotericin B, and flucytosine. However, all of these agents need to be administered intravenously. According to the database *drugs-porphyria.org*, fluconazole, itraconazole, and voriconazole are ‘probably porphyrinogenic’. Posaconazole is only ‘possibly porphyrinogenic’. From a clinical-pharmacological point of view, we recommend the continuation of caspofungin IV. However, if a switch to oral treatment is required, we consider posaconazole as a viable alternative to caspofungin IV.” ^56^	A plethora of drugs may trigger bouts of acute porphyria. Databases such as *drugs-porphyria.org* can help identify and avoid these agents in affected patients.
9	“A 57-year-old patient is being treated with mycophenolate mofetil as part of the immunosuppressive regimen following lung transplantation. What is the target range of the mycophenolate plasma level? The patient’s current trough plasma level is 14.4 mg/l.”	“Given the poor correlation between mycophenolate trough plasma levels and clinical outcome, reliable target ranges are not available. Additionally, different mycophenolate preparations show a high degree of variability in terms of plasma levels achieved. If TDM is indicated, the AUC 0–12 h (AUC_0–12_) is favored. Filler and colleagues have suggested the following approach: determination of mycophenolate plasma levels as trough concentration (C_0_), half an hour (C_0.5_), and two hours (C_2_) after mycophenolate intake and application of the following formula: AUC_0–12_ = 10.014 + 3.948 × C_0_ + 3.243 × C_0.5_ + 1.011 × C_2_. Better results can be obtained with an additional measurement after 4 h (C_4_) and utilization of the following formula: AUC_0–12_ = 8.217 + 3.163 × C_0_ + 0.994 × C_1_ + 1.334 × C_2_ + 4.183 × C_4_. According to Yakubi et al., the preferred AUC_0–12_ range lies between 22.8 and 40.5 μg × h/ml.” ^57–59^	TDM of mycophenolate mofetil represents a clinical-pharmacological challenge to physicians and is considerably more complex than conventional TDM. Several measurements of mycophenolate plasma levels after drug intake as well as the application of a multivariate linear regression equation are required.
10	“We would like to institute a pharmacological antidepressive therapy in a 28-year-old male patient. As the patient is additionally suffering from visual snow syndrome (a neurological disorder characterized by tiny flickering dots throughout the visual field), his neurologist warned him of SSRIs. Are there scientific data that justify this claim?”	“While the pathophysiology of visual snow syndrome remains mostly unknown, Eren and colleagues suggested a connection to serotonergic transmission. This hypothesis is substantiated by the partial therapeutic effect of lamotrigine, which exerts an inhibitory effect on 5-HT_3_ receptors. Considering ophthalmological ADRs of SSRIs (e.g., blurred vision), we advise against the use of SSRIs. Instead, we suggest mirtazapine. Mirtazapine has proven efficacy against depression and, to the best of our knowledge, no negative impact on visual snow syndrome has been reported.” ^60,61^	If pharmacological antidepressive therapy is indicated in a patient with visual snow syndrome, caution must be exercised to not deteriorate the symptoms of visual snow syndrome. If possible, SSRIs should be avoided.

ADR denotes adverse drug reaction, AUC area under the curve, BMI body mass index, CYP cytochrome P450, ECG electrocardiography, FGA first-generation antipsychotic, INR international normalized ratio, IU international unit, IV intravenous, no. number, PO per os, SC subcutaneous, SmPC summary of product characteristics, SSRI selective serotonin reuptake inhibitor, and TDM therapeutic drug monitoring.

## Discussion

In this study, we analyzed a total of 594 queries submitted to the clinical-pharmacological drug information center of Hannover Medical School. 82.8% of the queries were patient-specific; approximately one in three queries came from internists. However, when subdisciplines were evaluated separately, psychiatrists, urologists, and trauma surgeons represented the healthcare professionals who most frequently consulted the clinical-pharmacological DIC. ADRs, indications/contraindications, and PDIs were the three most frequently inquired query categories. As compared to general queries, patient-specific queries were statistically significantly more often related to ADRs, PDIs, and PKIs.

In their article from 1999, Schwarz and colleagues suggested that “the periodical analysis of the types and sources of enquiries may point to latent problems and needs in the medical community”, a notion we fully agree with. The most recent data from Germany on clinical pharmacologist-led DICs which provide DI to other healthcare professionals stem from the year 2000^26^. Therefore, updating this knowledge was urgently warranted.

A strength of the present analysis is the inclusion of nearly six hundred queries, which represents a case number of similar^2,12,15–17,25,27,28^ or higher magnitude compared to other studies in the field^8–11,14^. Furthermore, the structure of the clinical-pharmacological DIC of Hannover Medical School complies with recommendations such as a mixed staff of clinical pharmacologists and clinical pharmacists as core team^19^ and the involvement of physicians from other disciplines (e.g., internists, geriatricians, and psychiatrists) in terms of close professional cooperation and regular interdisciplinary exchange^19,29^.

The high degree of specialization at our university hospital may account for the large proportion of patient-specific queries (82.8%) as compared to general queries (17.2%), a finding that stands in contrast with reports by Almazrou et al.^30^ and Tefera et al.^15^ from pharmacist-led Saudi Arabian and Ethiopian DICs, respectively, where patient-specific and general queries were more evenly distributed (Almazrou et al. study: 44% patient-specific queries vs. 55% general queries; Tefera et al. study: 53.1% patient-specific queries vs. 46.9% general queries). While answers to general queries usually aim at delivering factual DI, responses to patient-specific queries focus on individualized treatment approaches and provide critical evaluations of the advantages and disadvantages of specific pharmacological therapies in distinct patient contexts, taking the patient’s age, sex, severity of the disease in question, clinically relevant comorbidities, and comedication into consideration. The delivery of factual DI—e.g., information on drug availability, drug stability, drug compatibility, adverse drug reactions, drug interactions, and so forth—is a core competence of pharmacists. Benefit–risk evaluations of pharmacological therapies within the individual patient context, on the other hand, are the mainstay of physicians. These professional differences may explain the discrepancies observed between the Almazrou et al. study^30^ or the Tefera et al. study^15^ (both pharmacist-led) and our investigation (physician-led). Of particular importance, it must be highlighted that both pharmacists and physicians play decisive roles in delivering DI and in taking care of pharmacotherapy safety. Pharmacists and physicians should not be regarded as competitors in these fields but—quite the opposite—they should be perceived as complementing each other with their specific expertise and competences.

In the present study, queries submitted by physicians dominated in comparison with queries from pharmacists (96.1% vs. 0.3%), which is in line with previous reports^9,14,17,22,25^, but in contrast to other studies^13,15,16,28,30^. The comparatively low proportion of queries submitted by pharmacists in our study may be owing to the fact that pharmacists in Germany have the opportunity to consult other institutions for DI, such as the DI services provided by the Chambers of Pharmacists (*Landesapothekerkammern*).

In the study by Schwarz et al., the primary users of a regional DIC in Dresden, Germany, were general practitioners, internists, and pediatricians^27^, as opposed to psychiatrists, urologists, and trauma surgeons in our analysis. However, if the different subdisciplines of internal medicine (e.g., rheumatology, pneumology, cardiology, and so forth) were aggregated, approximately one in three queries submitted to our clinical-pharmacological DIC came from internists. Intriguingly, we received a considerable proportion of queries (i.e., 14.0%) from surgeons, which contrasts with an older report by Tröger and Meyer from Magdeburg, Germany, in which surgical disciplines only played a minor role^26^. The high proportion of queries submitted to our clinical-pharmacological DIC by surgeons may reflect increasing interdisciplinary cooperation between conservative and surgical disciplines that has developed at Hannover Medical School in recent years. For example, besides operating the clinical-pharmacological DIC, our institute actively participates in the orthogeriatric co-management of older patients, together with trauma surgeons, internists, and geriatricians^31^.

Reppe and colleagues suggested that “the physical proximity of the [drug information] centers might lower the threshold [for enquirers] to initiate contact”^5^. Based on the results of our study, we can draw similar conclusions: many queries were received from departments with whom close professional cooperations exist, for example through interdisciplinary ward rounds with colleagues from psychiatry^29^ or trauma surgery^31^, and through the participation of our institute in the antimicrobial stewardship (AMS) program at Hannover Medical School^32^. Of note, personal contacts may lower the threshold to submit queries to the clinical-pharmacological DIC. Notwithstanding, queries from cooperating departments also have to be submitted via the regular route, that is, via e-mail or SAP.

Interestingly, the query category most frequently addressed by healthcare professionals in our study—i.e., ADRs—did not change between 1996^33^ and the current analysis. ADRs were also the query category most frequently addressed by 26.8% (22/82) of European DICs surveyed by Müllerová and Vlček in 1996^20^. The higher proportion of queries dealing with ADRs in our study (44.8%) as compared to other investigations (between 8.7 and 32.5%^2,8,9,15–17,21,26,27,34^) may be explained by the fact that our institute also houses the pharmacovigilance unit of Hannover Medical School and therefore possesses particular expertise in the evaluation of (serious) adverse events. According to a survey conducted by Müllerová and Vlček, only approximately one-third of European DICs are additionally engaged in pharmacovigilance activities^20^.

Müllerová and Vlček reported that approximately half of European DICs answered between 100 and 1000 queries per year^20^. Hence, the number of queries answered by our clinical-pharmacological DIC during the study period (i.e., 594 queries in three years and seven months) can be considered as low average. However, it must be taken into account that 43% (35/82) of the European DICs targeted their activities at healthcare professionals and the lay public^20^, whereas our clinical-pharmacological DIC is restricted to healthcare professionals working at a university hospital and academic teaching hospitals. Therefore, we assume that the complexity of queries submitted to our clinical-pharmacological DIC is considerably higher—on average—compared to queries submitted to the DICs investigated by Müllerová and Vlček^20^. This is exemplified by a selection of queries and corresponding answers (Table 3), which relate to rare and complex diseases such as mycosis fungoides (case no. 1), insulin autoimmune syndrome (case no. 2), periodic hypothermia (case no. 7), acute porphyria (case no. 8), and visual snow syndrome (case no. 10). In addition, temporal effects may play a role as queries to DICs have become increasingly complex in recent years^35^. For example, in a survey among DICs in the United States, 70% (46/66) of respondents described an increase in the number of complex DI queries between 2003 and 2008^18^.

To date, only five author groups have presented individual DI queries^6,10,11,21,33^, but only in abbreviated form^6,21^, or without provision of corresponding answers^10,11^. Apart from the Lumpe et al. article^33^ from 1998, our study is the only report to present and comment on a selection of clinically complex and intriguing DI queries so far, which can be considered a strength of our report.

PDIs (34.3% of all queries; 38.4% of patient-specific queries) and PKIs (29.6% of all queries; 31.5% of patient-specific queries) constituted frequent query categories in our study, in accordance with previous analyses by Alván et al., who stated that “questions concerning drug–drug interactions have increased steadily over the years, in parallel to a general increase in the number of drugs per patient, especially in the elderly”^23^. Our figures regarding drug–drug interactions were markedly higher than in a recent study by Tefera et al., who reported that only 5.9% of queries submitted to Gondar University Specialized Hospital DIC, Ethiopia, were related to drug interactions^15^. This discrepancy might be due to different patient populations, as patients in our study were considerably older (mean age 55.6 ± 22.9 years vs. 31.2 ± 20.1 years^15^) and presumably took more drugs on average, although no data are available to confirm this hypothesis.

Pharmacotherapy in older people is particularly complex as pharmacokinetics (e.g., reduced liver and kidney function) and pharmacodynamics (e.g., increased sensitivity to benzodiazepines) are significantly altered compared to younger individuals^36^. Approximately 6% of queries in our study were related to pharmacotherapy in older people. Previous studies, by contrast, did not investigate this topic as separate category^9,15,16,28,30,34,35^. The significant proportion of queries concerning pharmacotherapy in the elderly in our study may reflect the demographic development in Germany, which can be considered an aging society: to date, more than one-fifth of the German population is 65 years of age or older^37^, and this proportion is projected to rise over the coming decades^38^. Similar demographic trends are expected in emerging countries such as Brazil, China, and India^39^. Therefore, DICs in emerging countries might soon be facing a similar increase in the number of queries related to pharmacotherapy in the elderly. In addition, the comparatively high age of hospital patients as well as the participation of our institute in the orthogeriatric co-management must be taken into account as factors that might explain the sizable proportion of queries related to pharmacotherapy in the elderly in our study.

The importance of clinical pharmacologists in counseling on drug use during pregnancy was investigated by Erdeljić and co-workers, who demonstrated that clinical pharmacologists’ assessments were superior to the United States Food and Drug Administration (FDA) classification system in predicting pregnancy outcomes following pregnancy-related drug exposures^40^. Moreover, in a survey among physicians who consulted a Norwegian DIC on drug use during pregnancy, 9% of respondents stated that termination of a wanted pregnancy was avoided owing to the information provided by the DIC^24^. Overall, 92% of the respondents in this survey claimed that the answer from the DIC had a patient-specific clinical impact^24^. Surprisingly, only 3.5% of queries submitted to our clinical-pharmacological DIC during the study period referred to pregnancy or breastfeeding, which is significantly lower compared to older studies^2,27^, but in line with more recent reports^15^. The relatively low number of queries concerning pregnancy or lactation issued by healthcare professionals (88.6% (526/594) of whom were hospital-based) in our study is in accordance with previous investigations, which showed that hospital-based physicians less frequently inquire about these topics compared to primary healthcare physicians^27,33,41^. Moreover, the advent of freely accessible online databases such as Embryotox^42^, Bumps (Best Use of Medicines in Pregnancy)^43^, and Drugs and Lactation Database^44^ may explain the decline in the number of queries related to pregnancy or lactation compared to previous analyses^2,27^.

Of note, ADRs and drug–drug interactions (both PDIs and PKIs) were significantly more often addressed by patient-specific queries as compared to general queries in our study. This might be due to the fact that general information about ADRs and drug–drug interactions can easily be retrieved by physicians and other healthcare professionals themselves in SmPCs and drug interaction compendia (for example, via the drug information program AiDKlinik), without consultation of the clinical-pharmacological DIC.

Limitations of the present study mainly arise from its monocentric and retrospective design. Besides, we did not investigate the time spent answering queries and the references used in doing so. These topics have been covered comprehensively by other authors^2,9,11,12,15–17,22,25,27,34,45^. However, the most important limitation of the present study is that no information on patient-related outcomes was available. Hence, it was not possible to determine if consultation of the clinical-pharmacological DIC by treating physicians benefited their patients. To investigate this aspect, a control group would have been necessary, which was not feasible within the constraints of this study—a limitation our analysis shares with other reports^7–12,14–17,21,25–27,30,34^. Due to the exploratory character of our study, the statistical test results were not adjusted for multiple testing and must therefore be interpreted with circumspection.

Healthcare professionals usually do not have time resources to address time-consuming DI queries themselves^22^. Although the accessibility of scientific literature has been facilitated considerably over the past decades, primarily due to the advent of online search capabilities, the amount of available literature on a given pharmacological topic is substantially greater nowadays^22^. In addition, the complexity of DI queries has increased in recent years^22^. Therefore, the time spent on answering DI queries has not decreased significantly compared to former times^22^, suggesting an ongoing need for DICs to unburden clinicians in their daily routine.

Taken together, our study suggests that DI query characteristics depend on country-specific demographic factors (e.g., emerging vs. developed countries), the affiliation and catchment area of DICs, as well as on the composition of the DIC staff (clinical pharmacologist-led vs. pharmacist-led). We hypothesize that clinical pharmacologists and pharmacists can contribute effectively to a high quality of patient care and a high level of pharmacotherapy safety through DICs. Further studies are required to elucidate whether the utilization of DICs actually improves clinically relevant patient outcomes.

## Supplementary Information

Supplementary Information 1.

Supplementary Information 2.

## Data Availability

The data that support the findings of this study are available upon reasonable request from the corresponding author.

## Abbreviations

ADR: Adverse drug reaction
AMS: Antimicrobial stewardship
AUC: Area under the curve
BMI: Body mass index
Bumps: Best Use of Medicines in Pregnancy
CME: Continuing medical education
CPOE-CDSS: Computerized Provider Order Entry and Clinical Decision Support System
DI: Drug information
DIC: Drug information center
ECG: Electrocardiography
FDA: Food and Drug Administration
FGA: First-generation antipsychotic
INR: International normalized ratio
IQR: Interquartile range
IU: International unit
IV: Intravenous
No.: Number
PDI: Pharmacodynamic interaction
PIM: Potentially inappropriate medication for older people
PKI: Pharmacokinetic interaction
PO: Per os
SAP: Systems, Applications, and Products in Data Processing
SC: Subcutaneous
SD: Standard deviation
SmPC: Summary of product characteristics
SOP: Standard operating procedure
SSRI: Selective serotonin reuptake inhibitor
TDM: Therapeutic drug monitoring

## References

[1] Nova Manosalva MA, López Gutiérrez JJ, Cañas M (2016). Drug information centers: An overview to the concept. Rev. Colomb. Cienc. Quím. Farm..

[2] Llerena A, Öhman B, Alván G (1995). References used in a drug information centre. Eur. J. Clin. Pharmacol..

[3] Dombrowski SR, Visconti JA (1985). National audit of drug information centers. Am. J. Hosp. Pharm..

[4] Gabay MP (2017). The evolution of drug information centers and specialists. Hosp. Pharm..

[5] Reppe LA, Spigset O, Schjøtt J (2016). Drug information services today: Current role and future perspectives in rational drug therapy. Clin. Ther..

[6] Öhman B, Lyrvall H, Törnqvist E, Alván G, Sjöqvist F (1992). Clinical pharmacology and the provision of drug information. Eur. J. Clin. Pharmacol..

[7] Harish C (2021). Assessment of the impact of clinical pharmacology consultations provided to hospital clinicians from the drug information center—An outcome research in a developing country. J. Pharm. Pract..

[8] Jeevangi VM (2012). Assessment and evaluation of drug information service provided by pharmacy practice department based on the enquirer's perspective. Int. Res. J. Pharm..

[9] Puttegowda SKBK, Lakshminarayana SY, Madavasetty AN (2010). Assessing the pattern of drug information queries in a rural south Indian tertiary care teaching hospital. Malay. J. Pharm. Sci..

[10] Behera SK (2017). Drug information center as referral service in a South Indian tertiary care hospital. Int. J. Pharm. Investig..

[11] Garg G, Patil A, Kasudhan KS (2019). Naturalistic evaluation of pharmacotherapy consultations provided to hospital clinicians: A developing country's perspective. J. Pharm. Technol..

[12] Joshi MP (1997). University hospital-based drug information service in a developing country. Eur. J. Clin. Pharmacol..

[13] Dos Santos L, Winkler N, Dos Santos MA, Martinbiancho JK (2015). Description of medication errors detected at a drug information centre in Southern Brazil. Pharm. Pract. (Granada).

[14] Tumwikirize AW (2011). Use of a pilot drug information centre. Afr. Health Sci..

[15] Tefera YG, Gebresillassie BM, Ayele AA, Belay YB, Emiru YK (2019). The characteristics of drug information inquiries in an Ethiopian university hospital: A two-year observational study. Sci. Rep..

[16] Ashenef A, Reshid E, Yilma Z, Melaku T, Chane T (2018). Assessment of the use and status of new drug information centers in a developing country, Ethiopia: The case of public university hospital drug information centers. Biomed. Res. Int..

[17] Samuel F, Dawit H, Ashenef A (2014). Utilization of recently established drug information centers located in the public hospitals of Addis Ababa, Ethiopia: An assessment. Ther. Innov. Regul. Sci..

[18] Rosenberg JM, Schilit S, Nathan JP, Zerilli T, McGuire H (2009). Update on the status of 89 drug information centers in the United States. Am. J. Health. Syst. Pharm..

[19] Schjøtt J, Spigset O (2019). Drug information centres and their provision of decision support: The Scandinavian experience. J. Clin. Pharm. Ther..

[20] Müllerová H, Vlček J (1998). European drug information centres—Survey of activities. Pharm. World Sci..

[21] Alván G, Öhman B, Sjöqvist F (1983). Problem-oriented drug information: A clinical pharmacological service. Lancet.

[22] Reppe LA (2014). Factors associated with time consumption when answering drug-related queries to Scandinavian drug information centres: A multi-centre study. Eur. J. Clin. Pharmacol..

[23] Alván G (2013). The continuing challenge of providing drug information services to diminish the knowledge–practice gap in medical practice. Eur. J. Clin. Pharmacol..

[24] Frost Widnes SK, Schjøtt J (2009). Drug use in pregnancy—physicians' evaluation of quality and clinical impact of drug information centres. Eur. J. Clin. Pharmacol..

[25] Tukukino C, Wallerstedt SM (2019). Drug information centre queries and responses about drug interactions over 10 years—A descriptive analysis. Basic Clin. Pharmacol. Toxicol..

[26] Tröger U, Meyer FP (2000). The regional drug-therapy consultation service centre—A conception that has been serving patients and physicians alike for 30 years in Magdeburg (Germany). Eur. J. Clin. Pharmacol..

[27] Schwarz UI (1999). Regional drug information service. Int. J. Clin. Pharmacol. Ther..

[28] Cardoni AA, Thompson TJ (1978). Impact of drug information services on patient care. Am. J. Hosp. Pharm..

[29] Heck J (2021). The interdisciplinary psychiatric ward round—Evaluation of a pilot project. Psychopharmakotherapie.

[30] Almazrou DA, Ali S, Alzhrani JA (2017). Assessment of queries received by the Drug Information Center at King Saud Medical City. J. Pharm. Bioallied Sci..

[31] Heck J (2021). The interdisciplinary orthogeriatric ward round: Recommendations for the clinical routine. Unfallchirurg.

[32] Joean O (2022). Clinical and microbiological effects of an antimicrobial stewardship program in urology—A single center before-after study. Antibiotics (Basel).

[33] Lumpe M, Junker W, Frölich JC, Kirch W (1998). Ein aufgabengebiet der klinischen pharmakologie: Individualisierte arzneimittelinformation. Dtsch. Arztebl..

[34] Scala D (2001). Italian drug information centres: Benchmark report. Pharm. World Sci..

[35] Timpe EM, Motl SE (2005). Frequency and complexity of queries to an academic drug information center, 1995–2004. Am. J. Health. Syst. Pharm..

[36] Mangoni AA, Jackson SH (2004). Age-related changes in pharmacokinetics and pharmacodynamics: basic principles and practical applications. Br. J. Clin. Pharmacol..

[37] Rudolf H (2021). Reduction of potentially inappropriate medication in the elderly. Dtsch. Arztebl. Int..

[38] Mosshammer D, Haumann H, Morike K, Joos S (2016). Polypharmacy—An upward trend with unpredictable effects. Dtsch. Arztebl. Int..

[39] World Health Organization. *10 Facts on Ageing and Health*. https://www.who.int/news-room/fact-sheets/detail/10-facts-on-ageing-and-health. Accessed 16 May 2022. (2017).

[40] Erdeljić V, Francetić I, Makar-Ausperger K, Likić R, Radacić-Aumiler M (2010). Clinical pharmacology consultation: A better answer to safety issues of drug therapy during pregnancy?. Eur. J. Clin. Pharmacol..

[41] Mörike K, Boesen N, Mikus G, Schwab M (1998). Drug therapy information: A service provided by clinical pharmacologists for physicians in private practice and hospitals. Eur. J. Clin. Pharmacol..

[42] *Pharmakovigilanz- und Beratungszentrum für Embryonaltoxikologie Charité-Universitätsmedizin Berlin*. https://www.embryotox.de/. Accessed 24 May 2022 (2022).

[43] UK Teratology Information Service. *Bumps (Best Use of Medicines in Pregnancy)*. https://www.medicinesinpregnancy.org/. Accessed 24 May 2022 (2022).

[44] US National Library of Medicine. *Drugs and Lactation Database (LactMed)*. https://www.ncbi.nlm.nih.gov/books/NBK501922/. Accessed 24 May 2022 (2022).

[45] Reppe LA, Spigset O, Schjøtt J (2010). Which factors predict the time spent answering queries to a drug information centre?. Pharm. World Sci..

[46] Talpur R, Ward S, Apisarnthanarax N (2002). Optimizing bexarotene therapy for cutaneous T-cell lymphoma. J. Am. Acad. Dermatol..

[47] Censi S, Mian C, Betterle C (2018). Insulin autoimmune syndrome: From diagnosis to clinical management. Ann. Transl. Med..

[48] Roy-Lachapelle A, Solliec M, Bouchard MF (2017). Detection of cyanotoxins in algae dietary supplements. Toxins (Basel).

[49] Manali KM, Arunraj R, Ramakrishnan GS (2018). Development of sensitive and specific multiplex PCR method for the detection of microcystin producing cyanobacteria in spirulina food supplements. Food Sci. Biotechnol..

[50] Saussele T, Burk O, Blievernicht JK (2007). Selective induction of human hepatic cytochromes P450 2B6 and 3A4 by metamizole. Clin. Pharmacol. Ther..

[51] Bachmann F, Duthaler U, Meyer zuSchwabedissen HE (2021). Metamizole is a moderate cytochrome P450 inducer via the constitutive androstane receptor and a weak inhibitor of CYP1A2. Clin. Pharmacol. Ther..

[52] Holt S, Schmiedl S, Thürmann PA (2010). Potentially inappropriate medications in the elderly: The PRISCUS list. Dtsch. Arztebl. Int..

[53] Li L, Geraghty OC, Mehta Z (2017). Age-specific risks, severity, time course, and outcome of bleeding on long-term antiplatelet treatment after vascular events: A population-based cohort study. Lancet.

[54] *Pharmakovigilanz- und Beratungszentrum für Embryonaltoxikologie Charité-Universitätsmedizin Berlin. Embryotox*. https://www.embryotox.de/. Accessed 01 Feb 2019 (2019).

[55] Blondin NA (2014). Diagnosis and management of periodic hypothermia. Neurol. Clin. Pract..

[56] Porphyria Centre Sweden. *The Drug Database for Acute Porphyria*. http://www.drugs-porphyria.org/. Accessed 04 Jan 2021 (2021).

[57] Scheffert JL, Raza K (2014). Immunosuppression in lung transplantation. J. Thorac. Dis..

[58] Filler G (2004). Abbreviated mycophenolic acid AUC from C_0_, C_1_, C_2_, and C_4_ is preferable in children after renal transplantation on mycophenolate mofetil and tacrolimus therapy. Transplant Int..

[59] Yabuki H, Matsuda Y, Watanabe T (2020). Plasma mycophenolic acid concentration and the clinical outcome after lung transplantation. Clin. Transplant.

[60] Eren OE, Schöberl F, Schankin CJ (2021). Visual snow syndrome after start of citalopram-novel insights into underlying pathophysiology. Eur. J. Clin. Pharmacol..

[61] Eren O, Schankin CJ (2020). Mirtazapine for treatment of visual snow syndrome: A case series with insights into pathophysiology and therapy. Clin. Transl. Neurosci..

[62] de Castro, T., Beier, C., Terkamp, C.* et al*. Insulin autoimmune syndrome: A rare, but important differential diagnosis of hypoglycemia.* Internist (Berl)*** 63**(2), 217–220 (2022). 10.1007/s00108-021-01180-0 PMC8813699 34698875

