# Supplementary material for: Clinical-pharmacological drug information center of Hannover Medical School: experiences and analysis from a tertiary care university hospital
Source: Sci Rep. 2022 Nov 12;12:19409. doi: 10.1038/s41598-022-24005-y (PMC9653451; doi:10.1038/s41598-022-24005-y)
Supplement: Supplementary file 1 — Supplementary Information 1. [file 41598_2022_24005_MOESM1_ESM.docx]

**SUPPLEMENTARY TABLE 1** Specialization of healthcare professionals who submitted queries (n = 594) to the clinical-pharmacological drug information center of Hannover Medical School between October 2018 and April 2022.

| **Specialization of inquiring healthcare professionals** | **No. of submitted queries** | **%** |
| --- | --- | --- |
| Psychiatry | 63 | 10.6 |
| Urology | 55 | 9.3 |
| Trauma surgery | 50 | 8.4 |
| Internal medicine, not otherwise specified | 36 | 6.1 |
| Rheumatology | 33 | 5.6 |
| Pneumology | 32 | 5.4 |
| Gastroenterology, hepatology, and endocrinology | 27 | 4.5 |
| Pediatrics | 27 | 4.5 |
| Gynecology and obstetrics | 24 | 4.0 |
| Psychosomatic medicine | 22 | 3.7 |
| General practice | 19 | 3.2 |
| Nephrology | 19 | 3.2 |
| Dermatology | 18 | 3.0 |
| Radiology | 18 | 3.0 |
| Hematology and oncology | 16 | 2.7 |
| Neurology | 16 | 2.7 |
| Orthopedics | 9 | 1.5 |
| Cardiology | 8 | 1.3 |
| Center for Rare Diseases | 8 | 1.3 |
| Emergency department | 8 | 1.3 |
| Abdominal surgery | 6 | 1.0 |
| Anesthesiology | 6 | 1.0 |
| Cranio-maxillo-facial surgery | 6 | 1.0 |
| Ophthalmology | 6 | 1.0 |
| Antimicrobial Stewardship | 5 | 0.8 |
| Cardiothoracic, transplantation and vascular surgery | 5 | 0.8 |
| Immunology | 4 | 0.7 |
| Neurosurgery | 4 | 0.7 |
| Dentistry | 3 | 0.5 |
| Intensive care medicine | 3 | 0.5 |
| Otorhinolaryngology | 3 | 0.5 |
| Plastic, aesthetic, hand and reconstructive surgery | 3 | 0.5 |
| Forensic medicine | 2 | 0.3 |
| Infectiology | 2 | 0.3 |
| Pharmacy | 2 | 0.3 |
| Radiotherapy | 2 | 0.3 |
| Human genetics | 1 | 0.2 |
| Pain therapy | 1 | 0.2 |
| Pathology | 1 | 0.2 |
| Physical and rehabilitative medicine | 1 | 0.2 |
| Public health service | 1 | 0.2 |
| Virology | 1 | 0.2 |
| Not documented | 18 | 3.0 |
