# Supplementary material for: Clinical-pharmacological drug information center of Hannover Medical School: experiences and analysis from a tertiary care university hospital
Source: Sci Rep. 2022 Nov 12;12:19409. doi: 10.1038/s41598-022-24005-y (PMC9653451; doi:10.1038/s41598-022-24005-y)
Supplement: Supplementary file 2 — Supplementary Information 2. [file 41598_2022_24005_MOESM2_ESM.docx]

**SUPPLEMENTARY TABLE 2** Query categories, stratified by specialization of inquiring healthcare professionals (assignment of more than one category per query was possible)

| **Specialization of inquiring healthcare professionals^#^** | **Total no. of submitted queries** | **Adverse drug reaction—no. (%)** | **Indication/**  **contraindi­cation—no. (%)** | **Posology/dose adjustment (e.g., due to renal or hepatic insuffi­ciency)—no. (%)** | **Therapeutic drug monitoring—no. (%)** | **Pharmaco­genetics—no. (%)** | **Pharmaco­dynamic interaction—no. (%)** | **Pharmaco­kinetic interaction—no. (%)** | **Pregnancy and breast­feeding—no. (%)** | **Pharmaco­therapy in elderly people—no. (%)** | **Other—no. (%)** |
| --- | --- | --- | --- | --- | --- | --- | --- | --- | --- | --- | --- |
| Internal medicine | 185 | 92 (49.7) | 76 (41.1) | 37 (20.0) | 26 (14.1) | 10 (5.4) | 57 (30.8) | 64 (34.6) | 8 (4.3) | 1 (0.5) | 21 (11.4) |
| Psychiatry and psychosomatic medicine | 85 | 46 (54.1) | 51 (60.0) | 26 (30.6) | 10 (11.8) | 9 (10.6) | 23 (27.1) | 25 (29.4) | 1 (1.2) | 3 (3.5) | 7 (8.2) |
| Surgery | 83 | 33 (39.8) | 35 (42.2) | 22 (26.5) | 6 (7.2) | – | 37 (44.6) | 18 (21.7) | 2 (2.4) | 17 (20.5) | 18 (21.7) |
| Urology | 55 | 7 (12.7) | 20 (36.4) | 9 (16.4) | 3 (5.5) | – | 36 (65.5) | 19 (34.5) | – | 8 (14.5) | 3 (5.5) |
| Pediatrics | 27 | 13 (48.1) | 7 (25.9) | 7 (25.9) | 6 (22.2) | 2 (7.4) | 5 (18.5) | 9 (33.3) | 2 (7.4) | – | 6 (22.2) |
| Gynecology and obstetrics | 24 | 12 (50.0) | 8 (33.3) | 7 (29.2) | 3 (12.5) | – | 11 (45.8) | 11 (45.8) | 1 (4.2) | 1 (4.2) | – |
| Radiology and radiotherapy | 20 | 11 (55.0) | 10 (50.0) | 1 (5.0) | 1 (5.0) | 1 (5.0) | 12 (60.0) | 4 (20.0) | – | – | 1 (5.0) |
| General practice | 19 | 8 (42.1) | 8 (42.1) | 3 (15.8) | – | – | 3 (15.8) | 3 (15.8) | 2 (10.5) | 2 (10.5) | 4 (21.1) |
| Dermatology | 18 | 3 (16.7) | 10 (55.6) | 8 (44.4) | 2 (11.1) | – | 4 (22.2) | 6 (33.3) | 2 (11.1) | 1 (5.6) | 2 (11.1) |
| Neurology | 16 | 8 (50.0) | 8 (50.0) | 6 (37.5) | 2 (12.5) | 1 (6.3) | 3 (18.8) | 4 (25.0) | – | 1 (6.3) | 3 (18.8) |
| Miscellaneous | 44 | 22 (50.0) | 21 (47.7) | 12 (27.3) | 4 (9.1) | 1 (2.3) | 11 (25.0) | 8 (18.2) | 1 (2.3) | 2 (4.5) | 14 (31.8) |
| Not documented | 18 | 11 (61.1) | 3 (16.7) | – | – | – | 2 (11.1) | 5 (27.8) | 2 (11.1) | – | 5 (27.8) |

^#^Data presented in aggregated form (i.e., without differentiation between subdisciplines of internal medicine, surgery, etc.)
